# Asteroids® and Electrocardiograms: Proof of Concept of a Simulation for Task-Switching Training

**DOI:** 10.5811/westjem.2018.10.39722

**Published:** 2018-11-16

**Authors:** Farhad Aziz, Bryan Yeh, Geremiha Emerson, David P. Way, Christopher San Miguel, Andrew M. King

**Affiliations:** Ohio State University Wexner Medical Center, Department of Emergency Medicine, Columbus, Ohio

## Abstract

**Introduction:**

Emergency physicians are interrupted during patient care with such tasks as reading electrocardiograms (ECGs). This phenomenon is known as task-switching which may be a teachable skill. Our objective was to evaluate the potential of a video game for simulating the cognitive demands required of task-switching.

**Methods:**

Emergency medicine residents took a pretest on ECG interpretation and then a posttest while attending to a video game, Asteroids®.

**Results:**

The 35 residents (63%) who participated, scored worse on the ECG posttest then they did on the pretest (p<.001; effect size=1.14). There were no differences between genders or training level.

**Conclusion:**

Interpreting ECGs while playing the Asteroids® game significantly lowered ECG interpretation scores. This shows the potential of this activity for training residents in task-switching ability. The next phase of research will test whether ECG reading performance while task-switching improves with practice.

## BACKGROUND

Emergency physicians (EPs) are continuously interrupted during patient care.^1^ Chisholm et al. found that EPs are interrupted nearly 10 times per hour requiring frequent shifts of attention across tasks.^2^–^3^ How EPs respond to interruptions is just beginning to be better understood.^4^ One such interruption involves the screening of electrocardiograms (ECGs) for life-threatening pathologies.

Task-switching varies with regard to the cognitive load requirements (Figure).^5^–^6^ The ability to task-switch during patient care is something all EPs must learn.^7^–^8^ As they progress through training, more responsibility and larger patient loads require more frequent task-switching. A method for deliberate practice of task-switching is needed to improve performance.^9^

We designed our task-switching simulation “Asteroids® and ECGs” to simulate a situation that required considerable cognitive processing while patient care related tasks (such as ECG interpretation) are gradually introduced. Our goal was to acclimate learners to shifting attention across cognitively demanding tasks.^10^ Ultimately, our hope is that resident performance will improve through deliberate practice with task-switching.

## OBJECTIVES

We sought to evaluate a task-switching simulation between two cognitively demanding activities with the hope that practice with the simulation might reduce the time required to switch tasks.

## CURRICULAR DESIGN

This study was a one-group pretest-posttest design that compared resident performance on ECG interpretation with no distractions to their performance on ECG interpretation while engaged in the video game Asteroids®.

### Population

The subjects were residents from our emergency medicine (EM) and EM / internal medicine (EM/IM) programs (N=56).

### Measurement/Instrumentation

The ECGs included a normal sinus rhythm or one of these patterns of pathology.

- ST-elevation myocardial infarction (STEMI)
- Complete heart block
- Ventricular tachycardia
- S1Q3T3/R-heart strain
- Brugada
- Electrical alternans
- Sinus tachycardia
- Atrial fibrillation with rapid ventricular rate or response
- Inferior-posterior STEMI
- Wellen’s syndrome
- Right bundle branch block
- Mobitz II Heart Block

ECG interpretation experts reviewed each strip and labeled them with the pathology and rating of difficulty. The pretest and posttest were equated for level of difficulty. A survey specialist familiar with the project developed an evaluation questionnaire.

### Study Procedure

The study was conducted during October 2017 with residents rotating through this activity on conference days.

Asteroids® (available at: http://www.freeasteroids.org/) is a video game in which the object is to prevent the player’s “spaceship” from being destroyed by asteroids. The player avoids asteroids by “shooting” or dodging them and receives points for the each asteroid destroyed. Residents were allotted time to try the game. Then they had 15 minutes of uninterrupted time to read eight ECG strips, circle the abnormality, provide a diagnosis, and make a patient management decision.

After the pre-test, residents were instructed to start the video game and treat it as if it were an important clinical task. Once residents started playing, they were provided with ECG strips at random intervals throughout the 15 minutes of the exercise. As in the pretest, residents had to interpret the ECG strips; however under this condition they had to maintain the game. Subjects turned in score logs and completed a survey at the end.

### Scoring

ECG pre- and post-tests were scored by assigning one point for identifying the ECG abnormality, and one point for the correct triage decision. Points were summed and converted to a percentage. We also recorded the resident’s video game score.

### Data Analysis

We performed analyses using IBM SPSS.^11^ We first conducted a preliminary three-factor (2×2×3) analysis of variance (ANOVA) with one repeated measure: pre- and post-test score (TIME) by resident post-graduate year and gender. The subsequent significant effect of TIME was analyzed with a paired t-test and effect size.^12^

## IMPACT/EFFECTIVENESS

Of 46 eligible residents, 35 (76.1%) participated in the study. Sixty percent were women, and all levels of training were equally represented.

The ANOVA resulted in no significant main-effects or interactions involving gender or postgraduate year (PGY) Level (Table). However, we observed a significant and large TIME effect, suggesting that residents performed worse on the ECG test while task-switching with the video game than they did on the ECG pretest (pre-test mean=63.2, standard deviation [SD]=13.7; Asteroids® mean=47.7; SD=12.5; t= 6.04, degrees of freedom=34, p<.001; effect size=1.19).^13^

All residents said the game made ECG reading more difficult. Most (91.7%) thought task-switching was difficult and more than half (53%) thought they could improve with practice.

The results confirm that the video game served as an effective distractor, requiring a cognitive load to compel EM residents to spend significant amounts of time switching between playing Asteroids® and reading ECGs. Results were similar for residents of both genders and all levels of training. The Asteroids® game yielded substantially lower ECG reading scores for everyone, regardless of their Asteroids® score or experience with gaming. We interpreted this to indicate that the Asteroids® game served as an adequate distractor regardless of previous experience or skill in video gaming. Based on this finding, we believe that this video game could be used to create an inexpensive simulation to practice task-switching. We hope that with deliberate practice under these simulated conditions the residents’ task-switching improves, since cognitive psychology researchers have found evidence that multitasking is a trainable skill.^14^

## LIMITATIONS

We recognize that while the Asteroids® video game serves as a significant distractor to reading and interpreting ECGs, the cognitive load of actual clinical disruptions may result in more “time to switch tasks” when compared to playing a video game.

## CONCLUSION

Interpreting ECGs while playing the Asteroids® lowered ECG reading performance. Our goal was to show that this simulation could be used to improve resident’s task-switching performance.

## Figures and Tables

**Figure:**
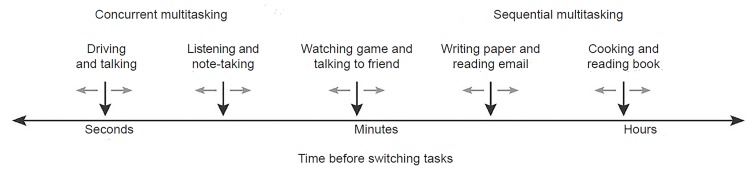
The multitasking continuum.^6^

**Table:** Results of a three factor (2×2×3) analysis of variance with one repeated measure involving 35 emergency medicine residents.

Source	df	Mean square	F-value	p-value
Between-subjects effects				
Gender	1	102.5	.909	.348
PGY	1	161.1	1.43	.256
Gender x PGY	2	226.3	2.00	.153
Error	29	112.8		
Within subjects effects				
Time	1	7890.9	35.07	.000*
Time x gender	1	27.0	.120	.732
Time x PGY	2	170.2	.756	.478
Time x gender x PGY	2	67.3	.299	.744
Error	29	225.0		

* Based on pretest mean and standard deviation of 63.2 (13.7) and posttest mean and standard deviation of 47.7 (12.4). Associated Cohen’s d effect size for the main effect of time within this repeated measures designs was: 1.07 (95% confidence interval [.52–1.52]).^11^

*PGY*, postgraduate year; *df*, degrees of freedom.
